# Lessons learned from twelve years of HIV Seroprevalence and Behavioral Epidemiology Risk Survey (SABERS) development and implementation among foreign militaries

**DOI:** 10.1371/journal.pone.0203718

**Published:** 2018-09-07

**Authors:** Stacy Endres-Dighe, Tonya Farris, Lauren Courtney

**Affiliations:** 1 Biostatistics and Epidemiology, RTI International, Rockville, MD, United States of America; 2 Biostatistics and Epidemiology, RTI International, Washington, DC, United States of America; Uniformed Services University of the Health Sciences, UNITED STATES

## Abstract

Circumstances within the military environment may place military personnel at increased risk of contracting sexually transmitted infections including HIV. Since 2005, RTI International has provided technical assistance to the Seroprevalence and Behavioral Epidemiology Risk Surveys (SABERS) program and supported the development and implementation of SABERS survey instruments in 18 countries. RTI staff collaborated with the Department of Defense HIV/AIDS Prevention Program and host country military and health care leadership to develop a fully tested, culturally appropriate survey and data collection instrument and build local capacity by identifying and training local interviewers. We summarize the critical steps, challenges faced, and lessons learned from 12 years’ experience developing, testing, and implementing SABERS instruments among military populations in Africa, Asia, and the Caribbean.

## Introduction

HIV is a leading cause of death globally [1] and an issue of major global public health concern. Circumstances within the military environment, such as high mobility, long periods away from home, disposable income availability, and increases in casual sexual relationships, may place military personnel at increased risk of contracting sexually transmitted infections (STIs) including HIV. Military personnel are also at elevated risk for occupational injuries, increasing the likelihood of HIV acquisition through contact with infected blood or via open wounds. A large proportion of soldiers are younger, susceptible to peer pressure [2,3], and fall within sexually active age groups. Militaries often report high rates of risky sexual behavior and low condom use among enlisted men and women [4–9], further highlighting the importance of targeted prevention efforts among these subgroups.

The military is a highly mobile population [10]. Extended, foreign deployment is common and results in exposure to biologically unfamiliar environments. During deployment, military personnel live and interact freely with the general population amplifying the risk of HIV contraction [7] and transmission [5, 6, 11]. HIV infection can impact the readiness of a military if skilled and experienced soldiers are lost to AIDS [12], and represents a national security threat with far reaching consequences [13, 14]; a fact made official by the United Nations Security Council in 2000. Reliable data on the spread of HIV and its risk factors is essential for an effective response to the HIV epidemic and resulting health, security and economic consequences. HIV bio-behavioral risk studies provide a critical source of data to estimate HIV/STI prevalence and identify risk factors, allowing prevention programs to maximize impact by focusing on the drivers of the epidemic.

The military is a unique population, and the very nature of military service poses several challenges that must be overcome to ensure the collection of sound, reliable survey data, including (1) fear of disobeying orders, (2) fear of stigma, (3) concern that disclosure of HIV status or risk behaviors (e.g., drug and alcohol use) will affect military employment, (4) mobility, and (5) obtaining participation of high-ranking officials. Social (e.g., literacy rates, computer skills) and cultural factors of the implementing country must also be considered when tailoring the survey instrument and determining method of administration.

**Fig 1:**
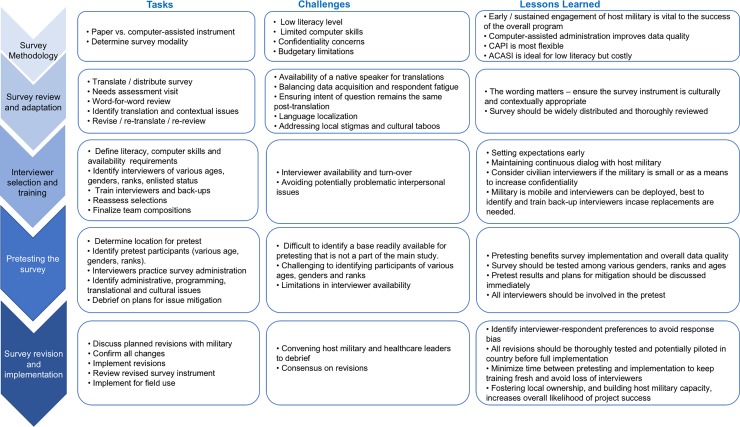
Tasks, challenges and lessons learned for SABERS survey development and implementation.

The U.S. President’s Emergency Plan for AIDS Relief (PEPFAR) was established in 2003. PEPFAR is the largest U.S. government initiative dedicated to a single disease and supports HIV/AIDS prevention, care, and treatment in developing countries [15]. The Department of Defense (DOD) HIV/AIDS Prevention Program (DHAPP) is an essential agency for implementing PEPFAR objectives and serves as the largest provider of HIV assistance among militaries worldwide. Seroprevalence and Behavioral Epidemiology Risk Surveys (SABERS) are a vital component in DHAPP activities [16]. SABERS are cross-sectional studies, implemented among foreign militaries, that consist of a survey to assess knowledge, attitudes, and behaviors related to HIV, coupled with rapid testing for HIV and other STIs. Since 2005, RTI has collaborated with DHAPP in providing technical assistance to SABERS in 18 countries. In addition to providing procedural design and management oversight, RTI supported the development and implementation of SABERS survey instruments. The following sections describe the key steps, challenges faced, and lessons learned from 12 years’ experience developing, testing, and implementing SABERS instruments among military populations in Africa, Asia, and the Caribbean (see **Fig 1**).

## Methods

### Survey methodology and administrative considerations

A necessary first step in the development and implementation of SABERS was to review data collection methodologies and determine whether a paper or computer-assisted instrument should be used. To inform this decision on a country-by-country basis, we sought input from host country military and health care leadership. We also considered the country context (e.g., socio-economic status, literacy levels, computer skill) and study budget. No matter the instrument used for data collection (paper or electronic) a critical next step was to determine which survey modality was culturally and contextually appropriate for the country of implementation. RTI supported the selection, development, and implementation of paper-assisted personal interviews (PAPIs) and three computer-assisted techniques used by the SABERS program including computer-assisted personal interview (CAPI), computer-assisted self-interview (CASI), and audio computer-assisted self-interview (ACASI). A description of all four survey modalities is provided in **Table 1**. Due to the sensitive nature of the data collected, and varying levels of military-required confidentiality, the names of the countries will not be revealed but instead referred to by region and year of implementation.

**Table 1:** Advantages and disadvantages for each survey modality.

Survey Mode	Description	Advantages	Disadvantages
PAPI	•Paper instrument •Face-to-face data collection •Questions read by interviewer •Responses written directly on instrument by participant or interviewer.	•Low cost •Low tech •No Internet required •No electricity required •No respondent literacy required (interviewer administered)	•Requires data entry of data from paper forms into a database •Prone to data entry errors •Complexity of navigating “skip patterns” and “data consistency checks” •Additional time for interviewer training and data collection •Delayed data analysis •Poorer data quality
CAPI	•Computer-based instrument •Face-to-face data collection •Questions read by interviewer •Responses entered directly into device by participant or interviewer	•Respondent literacy not required •No computer skill needed by respondents •Can ensure complete data collection •No additional data entry step •Skip patterns, data range checks, and data consistency checks can be programmed in, increasing data quality	•Respondent may fear judgment by interviewer •Respondent may fear breach of confidentiality •More expensive to procure computers •Support needed for computers •Gender of interviewer and respondent may be important
CASI	•Computer-based instrument •Self-administration •Questions read by respondent alone •Responses entered directly into device by participant	•Less response bias, increasing likelihood of “true” information •Can ensure complete data collection •No additional data entry step •Skip patterns, data range checks, and data consistency checks can be programmed in, increasing data quality	•Requires high level of respondent literacy •Requires computer skill •Support needed for computers •More expensive to procure computers
ACASI	•Computer-based instrument •Self-administration •Audio recording reads questions to respondent •Responses entered directly into device by participant	•Low literacy •Less response bias •Can ensure complete data collection •No additional data entry step •Skip patterns, data range checks, and data consistency checks can be programmed, increasing data quality	•Requires some literacy •Requires computer skill •Requires voice recordings of all questions and response options •More expensive •Support needed for computers •Takes longer to complete the survey

The advantages and disadvantages of each survey mode (described in **Table 1**) were reviewed with the host military to determine applicability. Literacy level, skill with computers, confidentiality concerns, and respondents fear of stigma, judgement preventing participation, or failure to provide honest survey responses were all discussed with local collaborators. We also considered interviewer and device availability, along with project budget, to determine the optimum survey methodology for use in each international setting.

### Survey review and adaptation

SABERS instruments averaged 116 questions in length and comprised 8–15 modules designed to capture the following types of data: demographic information, sexual history, condom use and accessibility, male circumcision, use of and access to HIV testing, gender-based violence, alcohol and drug use, posttraumatic stress, depression, HIV, tuberculosis, other STI knowledge, and care and treatment practices. A template survey, including questions from the modules described above, was developed and used as a starting point for opening SABERS review and adaptation. We translated the template into the country’s most commonly used language (as confirmed by the host military). The translated template was then distributed via e-mail to, and reviewed by, the host military to determine appropriateness of the selected modules. As described by Macera et al. [16], the host military makes an initial assessment of which modules were applicable and culturally appropriate and identified other important topical areas that were overlooked.

We conducted an initial in-country needs assessment visit, which served as an opportunity to (1) review the proposed survey instrument in detail and define required revisions, (2) access whether the proposed instrument is appropriate for the local military context and accepted by host country collaborators, and (3) establish a rapport between SABERS team members (DHAPP and RTI staff) and the host country’s military and health care leaders. During the needs assessment visit, the survey was reviewed, word for word, in its entirety to ensure that there were no translational or contextual issues. For example, there was typically not a direct translation for all terms used in the survey so the translator would use language localization to adapt the translation to the targeted culture. However, unless the translator had subject matter expertise, such as a background in public health or epidemiology, he or she would not be able to precisely convey all concepts across the language barrier. Although there may not be an exact translation, the word-for-word review and vetting with the host military ensured that the intent and meaning of each translated question remained the same. We subsequently used inputs received during the needs assessment visit to update and refine the survey instrument.

### Interviewer selection and training

Data collection staff were generally provided by the host military. Several factors were considered when selecting staff to fill the role of interviewer including technological skill, literacy, time commitments, rank, gender, and even military status. Host military ownership of the SABERS was integral to this process. By engaging military leadership at an early stage, and involving them in all decision-making processes, the SABERS team ensured host country investment in the study and that personnel with the appropriate qualities were identified.

Following interviewer selection, we scheduled a second in-country, or “pretest,” visit that served to (1) provide interviewers with extensive training on SABERS administration, (2) allow trained interviewers to practice using the data collection instruments, (3) identify and address problems with survey implementation, and (4) identify translational or adaptation problems prior to the start of the study. Pretest training began with a word-for-word review of the survey to ensure that the intent of each question was clear. Interviewers were thoroughly trained to avoid bias by standardizing survey administration and collecting data with complete objectivity. Participation in all SABERS was completely voluntary. Interviewers were trained to ensure participants fully understood the voluntary nature of the study and their right to refuse any question, or stop participation, at any time. Interviewers also needed to be intimately familiar with the survey, no matter if it was respondent- or interviewer-administered, to ensure that they were capable of offering assistance, as needed.

We then provided practical training on use of the survey instrument and included materials needed for data collection, and, if computer-assisted collection was being used, how to operate the tablet or computer, and launch and navigate through the application. Interviewers practiced data entry using real-world scenarios designed to familiarize them with the programmed skips and differing sets of questions that would be asked depending on participant characteristics and responses. For example, the questions asked would vary depending on the participant’s gender, sexual history, HIV status, and alcohol and drug use. The practical training ensured that interviewers were comfortable with the survey as a whole and with navigating through various scenarios that would be encountered in the field.

Data security, and the importance of treating all participants and information with the utmost confidentiality and respect, is especially important in the military setting and was a key theme throughout all training. Interviewers were taught the importance of keeping the tablets (or paper-based instruments) out of view of potential onlookers and in data collection team possession at all times, and given tips for avoiding damage (e.g., keep liquids away, avoiding the use of pencils or pens on the touch-screen). Training also included instructions for how to troubleshoot potential issues. For example, during CAPI or PAPI administration it was ideal to have a single room designated for each interviewer/participant pair. However, a sufficient number of rooms on the military base were often not available to accommodate this setup, so we gave data collection personnel tips (e.g., use partitions or conduct the interview outside under trees far enough away to allow privacy) on how to overcome this issue. SABERS were often implemented in low and middle-income countries (LMICs) where continuous power, and Internet connectivity, were common challenges that also had to be addressed.

### Pretesting the survey

Regardless of the tool or survey modality used, all SABERS instruments were first pretested. This served the dual purpose of (1) training interviewers to practice using the instrument for data collection and (2) identifying and addressing issues prior to survey implementation. Pretesting of the survey typically took place at the end of the pretest visit, or approximately 3 months prior to the start of data collection allowing sufficient time for instrument revision. The host military selected pretest participants who were typically stationed at a base not intended for data collection. A diverse group of military members were selected for participation including men and women of various ranks and education levels. To emulate field conditions, the pretest was implemented at a military base.

To identify survey implementation issues, we taught interviewers to make note of questions or words that seemed to cause confusion among participants (e.g., questions requiring clarification or that took a long time to answer), any consistency checks that were triggered, and any issues with modality (e.g., computer failure, programming issues, respondent literacy incompatibilities) that arose during the pretest. All trained interviewers were actively involved in the pretest. The pretest concluded with a debrief involving host military leadership, interviewers, and the SABERS team to discuss findings and determine necessary revisions.

### Survey revision and implementation for field use

Pretest results, and interviewer and military input, were reviewed by the SABERS team and used to inform revisions to the survey instrument. We addressed all programming, translational, and operational issues and prepared a final instrument for implementation.

## Results and lessons learned

### Survey methodology and administration

**Fig 2:**
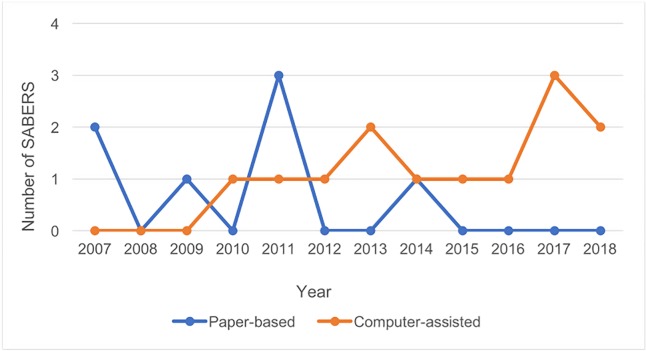
SABERS utilization of paper-based and computer-assisted technologies by year.

Paper-based methodologies have historically been the gold standard and were regularly used by SABERS projects up until 2009 (see **Fig 2**). The transition to computer-assisted technologies took over 4 years. There was a genuine lack of trust in electronic systems, and militaries were highly skeptical that these systems could improve confidentiality and increase data security. The general thought was that militaries could control hardcopy, paper-based surveys but that data on electronic devices could be confiscated without their knowledge. The first computer-aided SABERS was conducted in 2010 (see **Fig 2**) in Northeast Africa. The program quickly realized that computer-assisted technologies eliminated the need for data entry, saving time (shorter survey administration and overall data collection duration) and project funds. The use of programmed skip patterns in addition to range and consistency checks improved the quality and completeness of SABERS data. Entering data directly into a computer also increased safety and confidentiality (a common concern when implementing surveys in military settings) and offered project staff and host military collaborators the ability to proactively identify and resolve data issues.

Early computer-assisted SABERS used laptop computers, but the cost, size, and short battery life were a barrier for international use in LMICs. The advent of portable, handheld tablet computers, with small touch screens and extended battery life, further motivated the migration away from paper-based SABERS. Together, the confluence of this along with increased portability and decreased cost, improved the attractiveness and feasibility of implementing tablet-based SABERS in developing countries. After 2011, all but one SABERS was conducted using a computer-assisted technology (see **Fig 2**). In this instance, PAPI was the only instrument the Southeast Asian country would consider for the implementation of a SABERS in its military.

**Fig 3:**
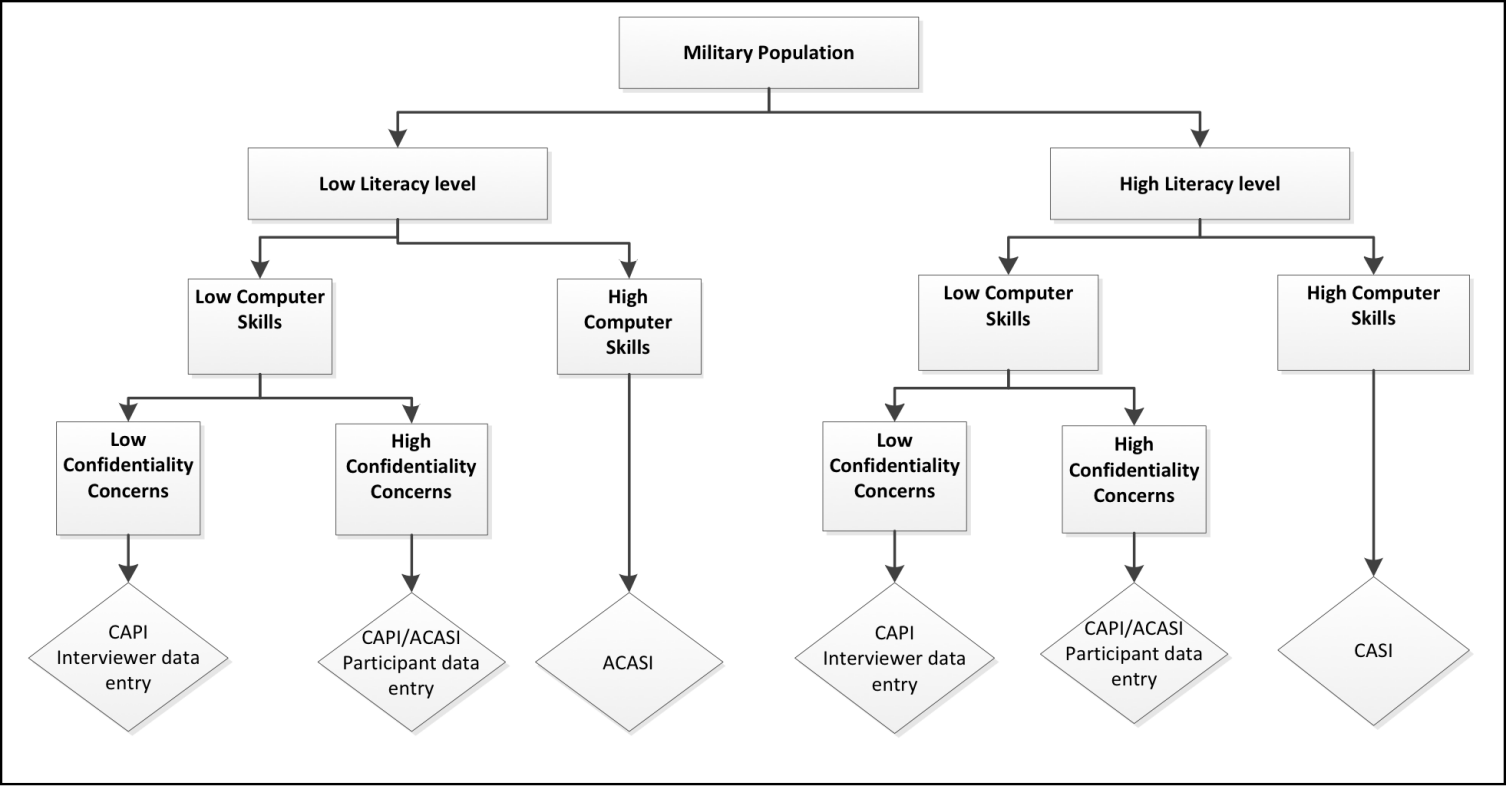
Factors to consider when determining the best survey mode.

Host country collaboration was integral to determining the best survey modality, as was engaging military leadership at an early stage. The advantages and disadvantages to each survey mode are described in **Table 1**. The majority of computer-assisted SABERS (54%) employed CAPI, followed by CASI and a combination of CAPI/ACASI, at 23% and 15%, respectively. Cost was a major deterrent to the use of ACASI. Aside from project finances, a number of factors (see **Fig 3**) must be considered while deciding on the survey modality to be used including host military literacy level, computer skill, and confidentiality and data security concerns. Militaries in Central America and West and Southeast Africa that had a high literacy level and computer skill opted to use CASI. A combination of CAPI/ACASI was employed in two SABERS conducted in East Africa where the military had low literacy and computer skills but an extreme concern about confidentiality of survey data and HIV test results. In both instances, participants were given the option of responding to sensitive questions (e.g., drug-use, sexual history, HIV status) using ACASI. However, to use ACASI the subjects had to pass three gateway questions to ensure that they possessed the minimal computer and literacy skills required for success with this system. Whatever the literacy level, CAPI served as a cost-effective tool for implementing SABERS in militaries with low computer skill.

### Survey review and adaptation

A keen understanding of the target audience was essential in the development of an effective survey instrument and collection of sound, reliable data. Survey review included careful consideration of the survey length, to avoid respondent fatigue, and all translations. Although there was not always an exact translation, the word-for-word review and vetting with host military ensured that the intent and meaning of each translated question remained the same. Accurate translation was especially important for SABERS using self-administration techniques because these methods lack interviewer assistance. For example, on one occasion the SABERS team was unable to identify a native Portuguese speaker to provide audio files for a SABERS using ACASI. A non-native translator was hired and her accent proved difficult for participants to understand. This one instance highlighted the importance of using a native speaker or translator, especially for SABERS using CASI or ACASI. The use of a native speaker for translations ensures language localization, that the correct terminology and dialect are employed, and ultimately that respondents understand the intent of each question.

Survey review often identified numerous contextual challenges. For example, the definition of “sex” was discussed in nearly all SABERS. “Sex” was often defined as “vaginal or anal sex between two willing individuals.” However, in one West African country the SABERS team learned that respondents would think that by saying “yes” they had sex, it would be indicative of having both anal and vaginal sex. In this instance, anal sex was so highly stigmatized that local counterparts encouraged its removal from the definition for fear that respondents would otherwise respond “no” to the question “have you ever had sex.” In general, both homosexuality and anal (including bisexual) sex were highly stigmatized topics or illegal to practice. Numerous militaries refused inclusion of these types cultural taboos all together. The sensitive nature of these topics, and the fact that SABERS were implemented in a work environment, highlighted the extreme importance of treating participants and their data with the utmost care and confidentiality. Defining types of sexual partners and delineating the differences (e.g., regular vs. casual) was also challenging. In numerous countries, a boyfriend or girlfriend was almost as strong a relationship as a spouse, so the wording of these questions required special consideration.

The module on alcohol usage also required careful review because the definition of both “alcohol” and what constitutes a “drink” varied tremendously from country to country. In one Southeast African country, a home-brewed beverage was such a staple source of nourishment that although it had a very high alcohol by volume ratio (ABV) it was not regularly identified as an alcoholic beverage. For this reason, the survey had to be revised so that the definition of an alcoholic drink included those brewed at home. Standardizing the definition of a “drink” was problematic because the volume of a beverage varied greatly as did the ABV.

Military ranks required careful review to ensure that all were correctly listed in the survey and available for respondent selection. Enlisted status was also frequently discussed. In one West African military, soldiers were considered enlisted but during the first year of service underwent training and thus did not see active duty. In this instance, to ensure that SABERS data were reflective of active duty military, the inclusion criteria were restricted to those who provided service greater than 1 year.

### Interviewer selection and training

Careful selection and thorough training of interviewers was instrumental in the successful development and implementation of SABERS. Interviewers were selected by the host military PI and comprised military (and occasionally civilians) of various genders, ages, and ranks. Civilians were often used if the host military did not have enough dedicated military staff for the study. Moreover, smaller countries preferred to use civilians as a means of promoting confidentiality by making participants feel more secure that their answers would not be part of military gossip. All interviewers were literate, and a basic level of computer skill was required of those involved in SABERS employing a computer-assisted technology. SABERS data collection phase ranged from 4 to 8 weeks, and team members were often deployed for extended periods. For this reason, continuous dialog with the host military was essential to ensure that the interviewers selected were available for the duration of data collection phase. The military also played a key role in assembling data collection teams that functioned well together and avoided potentially problematic interpersonal issues. We identified excess or backup interviewers who were trained to protect against turnover or if dismissal was required. Concerns about interviewer candidacy, including those who were consistently late, failed to take their role seriously, or lacked literacy or technical experience required to implement the survey, were raised with military leaders and, if necessary, a replacement was made.

### Pretesting the survey

The earlier the host military was engaged and actively involved the more effective and contextually appropriate the training. Pretest trainings typically ranged from 4 to 6 days. The length of training depended predominantly on the instrument being used and interviewer experience and skill. For example, paper-assisted administration required considerably longer training (4–5 days minimum) because interviewers needed to be intimately familiar with all skips, consistency checks, and survey content, whereas computer-assisted methods tended to require less training (3–4 days) because the skips and checks were programmed and worked independently of the interviewer. However, the instrument used did not always guarantee an expeditious training, as was the case for two SABERS that opted to use computer-assisted technology in a Southeast African country where little to no technology was available, and interviewers lacked the most basic computer skills.

Pretesting benefited survey administration because it enabled interviewers to practice using the instrument and become comfortable navigating through the survey before data collection officially began. Interviewers who were allowed to practice survey implementation were better prepared to identify and troubleshoot issues before they occurred in the field. For example, a military base would often not have sufficient space for interviewer-participant pairs to have a private room for survey administration. The pretest simulated this scenario and required interviewers to find an alternative setup (e.g., use of partitions, sitting in opposite corners of a large room or outside under individual trees) that would still guarantee respondent privacy. Pretesting also improved data quality by ensuring that the survey was correctly translated and adapted to the local setting. We examined participant-interviewer preferences. The preference for same-sex or opposite-sex pairings varied by country and military setting. For example, in one West African military the female pretest participants expressed a preference for a civilian, female interviewer because they were not a part of the military and less likely to know the participant or gossip. In other SABERS, male participants expressed a preference for male interviewers because they did not feel comfortable responding to some of the sensitive survey questions in the presence of a woman. Interviewer-respondent rank matching was also required for many SABERS. By working closely with the military, and identifying these potential challenges early, the SABERS team was able to address and prevent problems before they occurred in actual survey implementation.

### Survey revision and implementation

Survey implementation, translation, and modality issues were often identified during the pretest. Plans for addressing each issue were discussed by host military leadership, interviewers, and the SABERS team during the pretest debrief. The time it took to complete survey revisions and prepare for implementation depended largely on the modality used. For example, ACASI revisions required new written and audio translations and were considerably more time-intensive then CASI or CAPI. On average, it took 1 to 3 months after the pretest to revise the survey (e.g., translational problems), update programming (e.g., operational, consistency check, and skip issues) if computer-assisted technologies were used, and prepare the final instrument for implementation.

## Conclusions

The military is a unique population, and the very nature of military service and culture poses challenges that must be overcome to ensure collection of sound, reliable survey data. Host military involvement in every stage of SABERS survey development and implementation is vital to the success of the overall program. For this reason, the importance of the initial in-country needs assessment cannot be overstated because it opens the dialog, and solidifies the rapport, between SABERS team members and the host country military and health care leaders. Host military input on the country context (e.g., literacy level, computer skills, confidentiality concerns), along with study budget, should all be considered when determining the survey modality. Today, the benefits of using low-cost, highly portable tablet computers for data collection far outweigh those of paper in LMIC, a trend that is likely to continue over time.

It is essential to begin the survey review conversation early and establish a regular dialog, with host military counterparts, to refine the instrument. Wording matters! The incorrect use of words can offend respondents or introduce bias, so the use of a native speaker, with subject matter expertise for translations and audio recordings (if ACASI is used), is essential. A balance between data acquisition and survey length must also be maintained to avoid respondent fatigue. The survey should be widely distributed and thoroughly reviewed and tested among participants of various genders, ranks, and ages. Engagement of local counterparts will ensure that cultural taboos and stigmas are identified and addressed.

Interviewer selection and training is critical; survey data are only as good as the team collecting them. Local collaborators should be engaged to determine what interview-participant pairing is culturally appropriate (e.g., male-male, male-female, rankings, age, military vs. civilian), this information can also be used to inform selection of data collection team members. Data collection time commitments (e.g., length in the field) should be communicated in advance to ensure that the staff selected are available for the duration of the study. The military is a mobile population, with regular staff deployment, so it is ideal to identify and train backups who can serve as replacements if needed. Finally, no matter how many times you review the survey or data collection procedures, there will always be a surprise. The best way to overcome these issues is to foster local ownership and build capacity—thoroughly training and empowering data collection and military personnel will increase host military buy-in and success of the overall program.

Numerous programmatic (personal communication from Lauren Courtney, RTI International) and ethical (personal communication from Tonya Farris, RTI International) challenges were also faced and will be reviewed in two forthcoming companion papers.
